# A review on legal issues of medical robots

**DOI:** 10.1097/MD.0000000000038330

**Published:** 2024-05-24

**Authors:** Xiaoli Shentu

**Affiliations:** a Jiangsu University, Zhenjiang, China.

**Keywords:** artificial intelligence, law, medical robot

## Abstract

This paper examines the legal challenges associated with medical robots, including their legal status, liability in cases of malpractice, and concerns over patient data privacy and security. And this paper scrutinizes China’s nuanced response to these dilemmas. An analysis of Chinese judicial practices and legislative actions reveals that current denial of legal personality to AI at this stage is commendable. To effectively control the financial risks associated with medical robots, there is an urgent need for clear guidelines on responsibility allocation for medical accidents involving medical robots, the implementation of strict data protection laws, and the strengthening of industry standards and regulations.

## 1. Introduction

**Figure 1.**
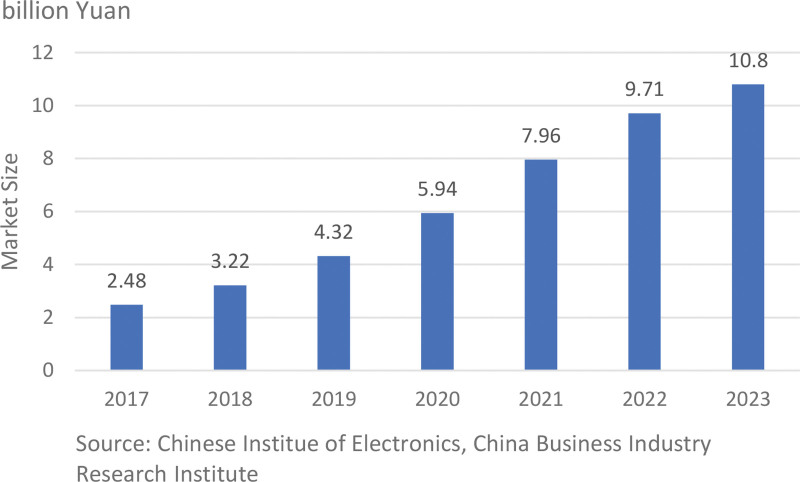
Market Size of the Medical Robot in China (2017–2023).

Looking at the evolution of artificial intelligence from a global perspective, the field of medical artificial intelligence (AI) emerges as a particularly prominent area of application. The 1980s saw the creation of the Quick Medical Reference by Randolph A. Miller at the University of Pittsburgh, marking an early milestone in medical AI. In 1984, the Laboratory of Computer Science at Massachusetts General Hospital initiated the DXplain project, which led to the release of its first version in 1986. A significant breakthrough came in 1997 when Dr Lars Edenbrandt and his coauthor Dr Bo Heden from Lund University Hospital in Sweden showed that artificial neural networks, a method based on computer technology, outperformed cardiologists in interpreting electrocardiograms.^[1]^ Over recent decades, the swift progress in medical robotics has transformed these tools into invaluable allies across various sectors of healthcare, including diagnosis, surgery, and rehabilitation. Although the start of medical robots in China was late, their development pace has been rapid. In recent years, the market size of medical robots has been growing increasingly larger, reaching 10.8 billion yuan in 2023, as shown in Figure 1 below.

Medical AI integrates multiple disciplines such as medicine, mechanics, and computer science, and is considered a new interdisciplinary field.^[2]^ Medical robots have significantly enhanced human health and welfare in unprecedented ways. For example, they have increased the precision in surgeries, accelerated diagnostic and treatment services, and made rehabilitation equipment more intelligent. Medical robots have not only reshaped the modern medical service landscape but also improved the effectiveness of medical services.^[3]^

However, the application of intelligent robots in the medical field also brings about numerous legal risks. The second part will introduce the potential legal risks associated with medical robots. The third part focuses on the legal issues presented in Chinese judicial practice, analyzing the basic stance of China on legal issues related to medical robots. The fourth part suggests corresponding measures for the prevention and control of legal risks associated with medical robots. The final part provides a summary.

## 2. The potential legal risk of medical robots

### 2.1. The legal status of medical robots

The key legal issue in the AI era is determining AI’s legal status. Modern intelligent robots differ from traditional machines by their ability to autonomously respond to situations like humans, without needing human commands. However, most legal experts agree that robots, despite their autonomy, remain machines and cannot be given the same legal status as humans.^[4]^

However, as robots become increasingly similar to humans in appearance and the gap in various aspects narrows, with their level of intelligence continuously improving, whether to grant legal personality to robots has become a controversial issue. Especially in some countries, animals have already acquired the legal status of “persons”^[5]^; modern society even constructs certain organizations as “juridical person.” Therefore, whether robot can become “person” in the legal sense is actually a decision made by legislators under different legal orders in different eras.

Over the past few decades, philosophers, scientists, and jurists around the world have engaged in intense debates on this issue. In 2017, Saudi Arabia granted citizenship to Sophia, a robot produced by Hanson Robotics, pushing the issue of legal personality for intelligent robots to a climax in discussions. Compared to this, the common law system is generally more open than the civil law system regarding the legal status of objects. For instance, Indian law recognizes temples and rivers as having the legal status of persons.^[6]^ It is conceivable that in such countries, arguing for the legal personality of an intelligent robot faces virtually no obstacles. However, civil law countries are more conservative on this issue. In 2016, the Legal Affairs Committee of the European Commission submitted a motion to the Commission, proposing that the most advanced autonomous robots be classified as “electronic persons.” In addition to granting these “electronic persons” specific rights and obligations, it also recommended the registration of intelligent autonomous robots.^[7]^ This effectively amounts to a form of civil legal personality. However, the European Union High-Level Expert Group on Artificial Intelligence does not agree.

The debate on the legal status of intelligent robots centers around 2 main viewpoints: the subjectivist approach, which supports recognizing intelligent robots as entities with legal personality similar to natural persons, and the objectivist approach, which refuses to grant them legal subject status, arguing they can’t be compared to humans. This issue is especially important for medical robots, given their advanced development and significant role in healthcare. Deciding whether to give medical robots legal subject status affects responsibility for issues like medical malpractice, data breaches, or criminal acts caused by these robots.

### 2.2. Legal risks of medical malpractice

The legal risks of medical malpractice refer to the liability that medical robots might incur for harming patients during medical procedures, leading to medical incidents. The technological imperfections of intelligent robots could be a primary cause of such incidents. For example, surgical robot systems on the market primarily rely on visual perception as their main feedback mechanism, yet doctors lack the fine tactile perception in formal scenarios when operating them. For rehabilitation and replication robots, small and lightweight drive units remain a major factor limiting the development of wearable robot systems. Rigid structural designs and heavier system weights could potentially cause secondary injuries to patients during use. However, these are stages that medical robot development must go through, and technology must advance through continuous practice.

Medical robots could have hidden design flaws or defects from their development and manufacturing that aren’t caught until they’re used in healthcare, potentially harming patients. Sometimes, these robots might malfunction due to new information they encounter during use, causing accidents. Identifying the cause of such incidents is challenging due to the complexity of medical AI’s mechanical structure and design. Pinpointing whether accidents stem from original design flaws, manufacturing errors, or issues arising during operation is difficult. If evidence shows a defect originates from the robot itself, then holding the creators or manufacturers responsible is fair, but proving this is practically difficult.

Holding research and development personnel accountable in the event of a medical accident is even more unreasonable. This is because the design and development of intelligent medical robots inherently involve unavoidable technical flaws. It is nearly impossible to expect programmers to be responsible for these kinds of technical defects unless it can be proven that the flaw was the result of malice. However, in most cases, designers can only predict possible risks of the program but would not intentionally create a robot that causes medical accidents. Under the influence of algorithmic “black boxes” and algorithmic bias, the autonomous decision-making capabilities of medical robots may exceed the designer’s intentions and original programming during operation, leading to unforeseeable harm. Such product liability cannot be attributed to the designers.

Thus, the question left for jurists is: Who should bear the responsibility?

### 2.3. Legal risks of medical data

The rapid development of AI, while providing convenience for patients to access medical care, also poses significant challenges to the protection of individual health data. Personal health data primarily includes basic patient information, diagnostic and treatment data, electronic medical records, medical imaging materials, and behavioral data throughout the diagnostic and treatment process. If these data are subjected to unauthorized access, leakage, or theft due to human or non-human factors, it can infringe upon the patient’s personal privacy, reputation, and safety.

During the research and development and testing phases of medical robots, developers need to collect a vast amount of data information, such as patients’ medical histories, family genetic histories, and diagnostic records. For example, Dr Lars Edenbrandt’s experiment collected over 10,000 patient electrocardiograms. In the use of medical robots, the intelligent system also needs to collect, process, and analyze various patient data to make quick and scientific medical judgments, perform correct operations during surgery, and provide accurate assistance during nursing and rehabilitation. Therefore, the groups that can access massive amounts of patient data include developers, distributors, medical staff, researchers, and of course, the medical robots themselves. At all stages of the development, testing, use, and disposal of medical robots, patients’ personal data are at risk of being leaked.

**Figure 2.**
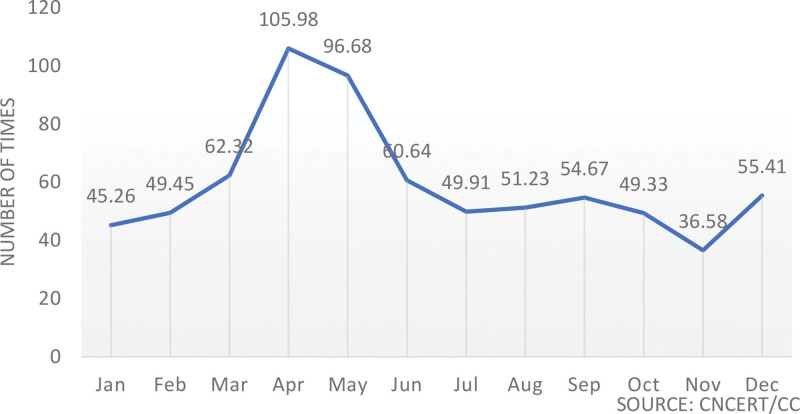
Statistical situation of the number of times biological genetic data exited the country by month in 2020.

It is well-known that the development of medical robots highly depends on original medical data. Only with the support of massive amounts of patient data can the continuous development of medical robots be advanced. It can be said that patient personal data make medical robots more intelligent, but the cost is an increasingly high risk of patient privacy breaches. For example, the “Artificial Intelligence Application Clinic” established by Sun Yat-sen University uses a cloud platform to assist clinical doctors in diagnosis and treatment. Patients’ examination data are synchronized to the cloud platform. Although this enables “expert-level” diagnosis and treatment, it also introduces significant data risks. According to statistics, in 2020 alone, more than 4.97 million medical imaging data instances from China were transmitted across borders through the internet, involving 3347 domestic IP addresses, with nearly 400,000 instances of data not being anonymized.^[8]^ In 2020, a total of over 7.17 million instances of domestic genetic data exiting the country via the internet were discovered, with monthly statistics shown in Figure 2. Therefore, the secure management of patient data is one of the important legal issues for medical robots.

### 2.4. Risk of criminal offenses

Whether medical intelligent robots can be considered as subjects of criminal liability, or whether robots should bear criminal responsibilities and be subject to criminal penalties, is a highly controversial issue. This controversy is closely related to the level of intelligence of medical robots and legal recognition of their status as subjects under the law.

Many jurists believe that the criminal law regulation of robot crimes is a fallacy, as legal scholarship tends to diminish the details of “autonomy in defining concepts within the technology sector, adhering instead to the so-called “technology neutrality or “technological reservation.”^[9]^ When the criminal intent of an intelligent robot can only be manifested as a piece of code or a set of programs, criminal law struggles to assess the subjective aspect of its crimes. Conversely, if a medical robot is utilized and controlled by a natural person to commit illegal and criminal activities, it is entirely possible to pursue the criminal liability of the natural person controlling the robot, without needing to discuss the issue of “robot” crimes at the criminal law level. Table 1 presents 3 scenarios of medical robot crimes:

**Table 1:** Criminal liability for medical robot crimes.

Type	Description	Potential criminal liability
Type 1 Human-Aided Crimes	Crimes where medical robots are used as tools by humans to commit offenses.	The human operator or accomplice would typically bear criminal liability.
Type 2 Programming Errors	Crimes resulting from flaws or errors in a medical robot’s programming.	Liability could fall on the programmer or the entity responsible for the robot’s maintenance, depending on the nature of the error and its foreseeability.
		If the error is unavoidable or unforeseeable, then the criminal should be identified as the medical robot. If the law does not grant robots legal subject status, then there is no guilt.
Type 3 Autonomous Decision-Making	Crimes committed by robots making independent decisions without direct human input.	Medical Robot should bear the criminal liability. If the law does not grant robots legal subject status, then there is no guilt.

The first category involves medical robots used or controlled by natural persons to commit crimes. In these cases, the robot is essentially just a “criminal tool” of the natural person, albeit a more advanced and intelligent tool compared to knives or guns. There is no controversy regarding criminal liability in such situations, and there’s no need to discuss the robot’s criminal liability. Criminal law only needs to penalize the controller of the robot.

The second category is where medical robots commit criminal acts due to system program malfunctions or defects. The criminal liability in these cases depends on the legal subject status of the medical robot. If the medical robot is anthropomorphized, meaning it is given a personality status similar to humans, then the robot would need to bear liability for medical negligence, and in cases of gross negligence, it would need to bear criminal liability for negligent crimes. If medical robots are not recognized as having an independent personality, then they remain as auxiliary tools for medical personnel, and the medical institution might bear tort liability in the civil domain.

The third scenario involves intelligent medical robots autonomously committing criminal acts, i.e., strong AI robots with the capacity for decision-making and thinking, which develop the intent to commit crimes during their operation. The classification of such crimes also depends on the legal recognition of the medical robot’s subject status. If they possess an independent legal personality, then the medical robot would have to bear criminal liability. If the law does not recognize the robot’s independent personality, then determining criminal liability becomes difficult.

## 3. Analysis of Chinese judicial situation involving medical robots

This section will analyze specific judgments involving medical robots in Chinese judicial practice. In the publicly available judicial decisions, there are not many judgments related to medical robots. However, from the limited judgments available, we can observe the legal practice status and specific practice claims regarding medical robots in China.

### 3.1. Judicial practice statistics

**Figure 3.**
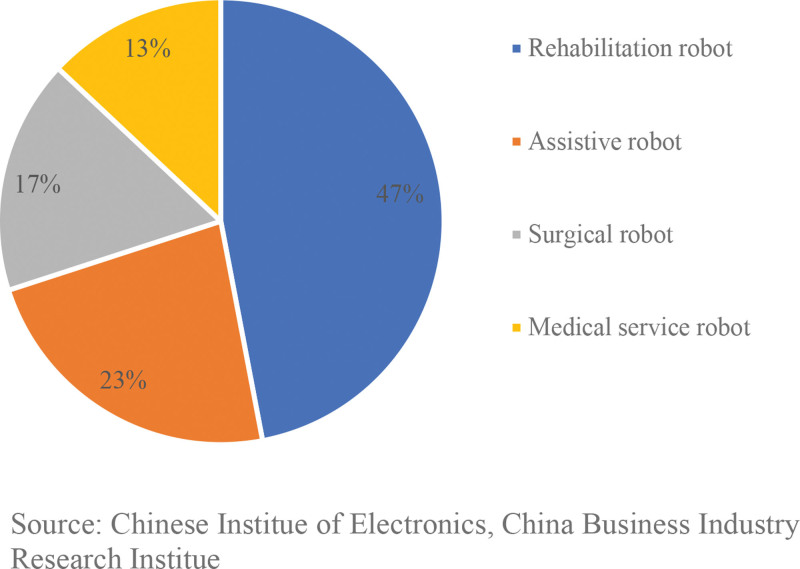
Market share distribution of the medical robot market in China.

In China’s medical robot market, rehabilitation robots account for the largest share at 47%, followed by assistive robots with a 23% share, while surgical robots and medical service robots have shares of 17% and 13%, respectively. Refer to Figure 3 below.

Although the market share of surgical robots is not high, the legal disputes arising from the use of surgical robots are the most numerous. Surgical robots, as innovative intelligent medical devices, can perform precise surgical operations in human body cavities, blood vessels, and densely nerve-packed areas. They offer advantages such as accurate positioning, minimal surgical trauma, low risk of infection, and rapid postoperative recovery. From 2018 to 2023, the Chinese surgical robot market grew from 1.07 billion yuan to 7.17 billion yuan and is expected to continue growing in the future.

As an example, using the Alpha Legal Intelligence Operating System (https://alphalawyer.cn/), a search was conducted with the keywords “medical,” “surgery,” and “robot.” After organizing the search results, a total of 33 relevant judgments involving medical robots were obtained. The vast majority of legal disputes were related to medical malpractice disputes arising from robot-assisted surgeries, with a few involving intellectual property disputes and insurance contract disputes. For detailed information, see Table 2:

**Table 2:** Surgical robot case situations.

Types of disputes	Number of cases
Medical malpractice disputes	22
Insurance contract disputes	7
Intellectual property disputes	4

From the aforementioned cases, it is evident that intelligent robots are extensively involved in surgical operations in China, such as robot-assisted laparoscopic left ureteral reimplantation, robot-assisted laparoscopic partial cystectomy, Da Vinci robotic right upper lobectomy and lymph node dissection, robot-assisted laparoscopic radical prostatectomy, left ureteral stent placement, robot-assisted distal pancreatoduodenectomy, Da Vinci robot-assisted thoracoscopic mediastinal surgery, robot-assisted extensive total hysterectomy with bilateral salpingo-oophorectomy, and robot-assisted closed reduction and cannulated screw internal fixation treatment, among others.

In these medical malpractice disputes, the reasons for litigation are mostly due to patients suffering physical harm after robot-assisted surgery, poor recovery outcomes, or other adverse consequences, leading patients to claim for tort compensation. Some patients question the qualifications of hospitals to perform robot-assisted surgeries, believing that the hospitals lack the credentials to conduct such surgeries, yet proceed to do so, resulting in physical harm. Additionally, some patients argue that the hospital did not inform them of the various risks associated with robot-assisted surgery or proceeded without their consent.

In insurance contract disputes, the main point of contention is whether the costs arising from robot-assisted surgery are covered under commercial insurance policies. In intellectual property disputes, the focus is primarily on issues of trademark and patent infringement related to medical robots.

### 3.2. Analysis of judicial practice

From the content of court judgments involving medical robots, the following conclusions can be drawn:

First, intelligent robots are already widely used in medical field, with surgical robots performing a variety of operations on patients. However, in specific cases, the level of intelligence and the degree of assistance provided by surgical robots are not the focus of current judicial decisions. It is generally considered that robot-assisted surgery is merely a method of surgery or an auxiliary tool for the surgeon, which does not imply that the robot itself is the agent conducting the surgery. Therefore, patients make tort claims against the hospital rather than seeking compensation from the developers of the surgical robot.

Second, medical robots do not possess independent legal personality under Chinese law. This view is consistent with the current legal regulations on the classification and management of AI products in China. For example, the “Guidelines for Artificial Intelligence Safety and Rule of Law (2019)” explicitly state: “Artificial intelligence derivatives do not qualify as legal subjects; their nature is that of tools, not independent entities.” Therefore, in judicial practice, intelligent robots in the medical field, regardless of the role they play in surgery, are explicitly classified as medical devices, i.e., tools used by doctors to treat diseases. This is different from legal practices abroad. For instance, when the Watson medical AI system in the United States caused harm to patients, it was deemed by the court as an “employee” rather than a medical device, with the hospital bearing vicarious liability for the damages caused.^[10]^ However, in Chinese judicial practice, damages caused by intelligent robots are considered as part of the fault in the medical process by doctors or hospitals, with the hospital being the direct subject of liability for compensation.

Third, judicial practice faces challenges in assessing medical negligence caused by medical robots. In medical malpractice disputes, third-party appraisal institutions are responsible for determining whether there was fault in the medical action and whether there is a causal relationship between the medical action and the patient’s injury. There have been instances where judicial appraisal institutions, due to the cutting-edge technology of surgical robots exceeding their appraisal capabilities, have issued letters of withdrawal.^[10]^ In the vast majority of cases, even if appraisal institutions provide an appraisal opinion, they do not comment on the program or level of intelligence of the medical robots, nor do they issue opinions on the causal relationship between the robot and the patient’s injury. Typically, the fault level of the medical institution is judged based on the entire medical action itself, including whether the treating physician’s choice to use a robot for surgery was reasonable.

Fourth, practice has shown that the use of strong AI medical robots is still in its initial stages, so the risk of criminal offenses involving medical robots is relatively low.

## 4. Measures to address legal risks of medical robots

Theoretical discussions on risks are more in-depth than practical risk considerations, but legal risks at the practical level are more complex and varied. The perfection of AI technology is certainly crucial, but the improvement of legal norms is an important safeguard for the continuous progress and development of medical robots.

### 4.1. Clarify the standards of liability

The medical field is characterized by strong professional barriers, and the technology of intelligent robots is still in the development stage. Globally, medical disputes caused by medical robots are showing an upward trend. Therefore, it is necessary to further clarify from a legal perspective who bears legal responsibility and how to allocate responsibility when medical malpractice is caused by medical robots.

Based on current medical practices, the assistive role of medical robots is greater than their autonomy, and there is still a significant gap between them and strong AI with autonomous consciousness. Therefore, treating medical robots as “tools” is more in line with the actual situation. Of course, if the functions of medical robots become increasingly powerful and their autonomy increases in the future, the possibility of granting them legal subject status cannot be ruled out. For now, it is more appropriate for medical robots to be used as medical assistive tools. If damages are caused by medical robots during the diagnostic and treatment process, they should be addressed according to the provisions on tort liability in the Civil Code of the People’s Republic of China.^[11]^

On this basis, it is also necessary to clarify the standards for judging liability in medical harm cases and establish a reasonable mechanism for sharing and responding to liabilities. When medical robots cause tortious harm, imposing excessive liability on medical institutions or the developers of medical robots could increase the burden on medical personnel and researchers, which is not conducive to the long-term development of AI. However, if accountability for related personnel is too lenient, or if patients harmed by medical robots cannot receive appropriate compensation, it would disregard the legal interests of citizens’ lives and health and represent an irresponsible legal direction. Therefore, legislators need to seek a balance between the rational values and technological rationality of AI.

At the same time, judicial appraisal institutions should also have corresponding measures to deal with appraisals involving medical robots. If appraisal institutions are unable to evaluate the tortious harm caused by medical robots, it becomes difficult to assign specific liability, making it even harder to establish a reasonable compensation system.

### 4.2. Improvement of data protection regulations

Regardless of whether it involves strong AI or weak AI, whether the medical robots serve an auxiliary role or play a leading role in medical procedures, the protection of patient data and privacy is a critical issue that must be addressed at this stage. In response to this, the European Union introduced the General Data Protection Regulation in 2018. This regulation is one of the strictest in terms of personal data protection and imposes the most severe penalties. It is also the most closely watched legal document on personal information protection globally over the past 2 decades.

To strengthen data protection, China has also introduced a series of legal norms to enhance the protection of patient data in the medical field. In 2010, the Ministry of Health issued the “Management Measures for Electronic Authentication Services in the Health System (Trial)” and the “Health System Electronic Certification Service System Series of Specifications.” In 2014, the National Health and Family Planning Commission released the “Management Measures for Population Health Information (Trial).” In 2016, the State Council issued the “Guiding Opinions on Promoting and Regulating the Development of Health and Medical Big Data Applications.” The promulgation of the “Personal Information Protection Law of the People’s Republic of China” in 2021 marks a new step forward in the protection of personal information in China.

The effectiveness of the regulations in genuinely protecting patient personal information remains a subject for further exploration. That is, the safeguarding of patient data and privacy necessitates not just the establishment of legal norms but also their effective execution in practice. Furthermore, there is a pressing need for more precise definitions regarding the categorization and protection levels of personal medical data, as well as a clear delineation of behaviors that violate patient privacy and the legal responsibilities associated with data breaches.

### 4.3. Strengthening the industry regulation

Practice has shown that China’s regulation of medical AI in all aspects is significantly lacking. Although the exploration of medical AI in China started relatively late, its development has been rapid, leading to a weakening in regulatory intensity. The regulation of medical AI requires the coordinated collaboration of producers, medical workers, medical institutions, the government, and the public.

Distinct responsibilities should be assigned to various stakeholders involved in regulating medical robots, necessitating a detailed approach towards the regulation targets, scope, and methodologies to establish an all-encompassing supervision and management framework. During the research and development phase of medical robots, it is imperative for designers to meld the unique aspects of the medical domain with ethical design principles, producing robots that comply with ethical norms. Embracing a philosophy of “responsible innovation” is crucial to ensure the meticulous development of medical robots. It is the duty of national science and technology departments to anticipate and evaluate the unforeseen impacts and results of research and innovation, guided by ethical consideration principles and the fundamental values of humanity.^[12]^ This approach guarantees that intelligent robots can make ethically aligned decisions and actions, effectively tackling issues of ethical responsibility. Only those medical robots that have undergone rigorous approval and assessment processes, conforming to established industry standards and requirements, should be put into operation.

Medical institutions and medical personnel must assume a “high scrutiny regulatory duty” in the application of intelligent medical robots with high-risk characteristics. At the current stage, medical robots do not possess legal subject status, and most medical robots serve as auxiliary tools for medical staff. Therefore, decisions regarding whether to use medical robots, when to use them, how to use them, and how to operate them during use all depend on the medical personnel and institutions.

## 5. Conclusion and future outlook

The journey of AI in the realm of medicine showcases a remarkable evolution. These advancements have not only paved the way for groundbreaking applications in diagnosis, surgery, and rehabilitation but have also introduced complex legal challenges. The discourse surrounding the legal status of medical robots, their potential to cause medical malpractice, breach personal data, and even commit crimes, underscores the need for a robust legal framework that evolves alongside technological progress.

The Chinese judicial perspective provides insightful reflections on these issues, revealing a cautious approach towards granting legal personality to AI and emphasizing the role of medical robots as tools rather than independent entities. This stance, while pragmatic, highlights the pressing need for clear liability standards, enhanced data protection measures, and stringent industry regulation to navigate the intricacies of medical AI applications.

Looking ahead, the dynamic interplay between technology and law necessitates ongoing dialogue and collaboration among all stakeholders involved in the development and deployment of medical robots. As we venture further into this uncharted territory, the ultimate goal remains to harness the full potential of medical AI while safeguarding patient welfare and upholding ethical standards. The path forward calls for “responsible innovation,” where advancements in medical technology are matched with equally progressive legal and ethical considerations, ensuring a future where medical robots continue to serve as invaluable allies in healthcare, within a framework that respects and protects human dignity and rights.

## Author contributions

**Writing – original draft:** Xiaoli Shentu.

**Writing – review & editing:** Xiaoli Shentu.
